# Plasma Applications in Biomedicine: A Groundbreaking Intersection between Physics and Life Sciences

**DOI:** 10.3390/biomedicines12051029

**Published:** 2024-05-07

**Authors:** Christoph V. Suschek

**Affiliations:** Department for Orthopedics and Trauma Surgery, Medical Faculty, Heinrich Heine University Düsseldorf, Moorenstraße 5, 40225 Düsseldorf, Germany; suschek@hhu.de

Plasma applications in biomedicine represent a groundbreaking intersection between physics and life sciences, unveiling novel approaches to disease treatment and tissue regeneration [1]. The journey of plasma medicine begins with a profound understanding of the interactions between cold atmospheric plasmas (CAPs) and biological entities. Initially conceived as a tool for material processing, plasmas have emerged as multifaceted actors, engaging in intricate dialogues with living cells and tissues. The novelty of this field lies not only in its capacity to disinfect and heal but also in its potential to unravel the mysteries of cellular responses to controlled plasma exposure [2,3,4]. Plasma devices operating under atmospheric pressure and at low temperatures offer a compelling avenue for treating fragile materials and living tissues without inducing thermal damage [2]. Recently, CAP has garnered increasing attention in healthcare, particularly within the realms of dermatology and surgery. CAP demonstrates therapeutic potential in addressing bacterial infections, impaired wound healing, and chronic wounds among patients [5]. Notably, CAP exhibits antibacterial properties capable of combating antibiotic-resistant strains, such as methicillin-resistant Staphylococcus aureus (MRSA), while preserving adjacent tissue integrity [6]. Furthermore, studies have elucidated CAP’s ability to modulate wound healing processes regulating key molecular pathways [7].

Research pertaining to the medical applications of cold plasma commonly falls under two principal categories: direct plasma treatment and indirect plasma treatment [3,8]. Direct treatment methodologies involve the treated tissue serving as an electrode interface, facilitating plasma generation between its surface and the electrode of the plasma device, typically utilizing dielectric barrier discharge (DBD) mechanisms. Conversely, indirect treatment methodologies entail plasma ignition within a conduit housing a flowing process gas, such as argon, helium, or air, thereby facilitating the conveyance of active species to the target substrate. Various plasma delivery systems, including plasma torches, plasma jets, plasma needles, and plasma pencils, have been developed and deployed in clinical settings.

In general, cold plasma systems engender a complex milieu of radical species, UV radiation, and charge carriers, each capable of modulating biological responses upon direct exposure to tissues or cells. When operated with ambient air, cold plasma systems yield substantial quantities of reactive oxygen species, encompassing ozone (O_3_), superoxide (O_2_^−^), and hydroxyl radicals (^.^OH), alongside reactive nitrogen species (RNS), such as nitric oxide (NO) and nitrogen dioxide (NO_2_) [9,10]. These redox-active species are postulated to underlie most CAP-mediated effects on cellular, tissue, and microbial physiology. Notably, in biological contexts, living cells and tissues typically exhibit a hydrated state or are immersed within a liquid milieu. Consequently, CAP interactions primarily transpire within this liquid environment, diverging from conventional dry plasma applications such as surface sterilization [11,12].

This Special Issue of Biomedicine, “*Plasma Applications in* Biomedicine”, brings together cutting-edge research addressing various aspects of this multidisciplinary field. This collection of articles delves into the potential therapeutic applications of CAPs and plasma-activated liquids (PALs) across diverse medical domains [12,13]. As we explore the diverse landscapes of plasma applications in biomedicine, it is essential to acknowledge the depth and breadth of ongoing research in this field. The presented works, within the confines of this Special Issue, serve as glimpses into the expansive potential of CAPs and PALs, echoing the broader literature. The intersection of physics and medicine, epitomized by plasma medicine, holds promise not only for addressing current healthcare challenges but also for unlocking new frontiers in diagnostics, treatment, and our understanding of the complex interplay between plasmas and living organisms.

The manuscripts featured in this Special Issue showcase the versatility of plasma technology, highlighting its role in addressing challenging medical scenarios. As we navigate through the abstracts, the diverse applications of CAPs and PALs become apparent, ranging from endodontics and recurrent aphthous stomatitis to wound healing, tissue regeneration, cancer treatment, and even antibacterial solutions for bone infection and vaginal health.

In “Non-Thermal Atmospheric Pressure Plasma Application in Endodontics”, Ana Bessa Muniz et al. conduct a comprehensive literature review, emphasizing the potential of non-thermal atmospheric pressure plasma (NTPP) in endodontics. The antimicrobial efficacy of NTPP against key endodontic microorganisms is explored, revealing promising results for clinical practice [14].

Norma Guadalupe Ibáñez-Mancera et al. present a pilot study on the “Healing of Recurrent Aphthous Stomatitis by Non-Thermal Plasma”. Their research demonstrates the effectiveness of NTP in reducing pain and inflammation and promoting rapid tissue regeneration in patients with recurrent aphthous stomatitis [15].

Moving beyond oral health, Lilith Elmore et al. investigate the “Healing Potential of J-Plasma Scalpel-Created Surgical Incisions”. Their study compares the outcomes of incisions made by a plasma scalpel with those created by a steel scalpel, revealing no significant difference in the appearance or physiology of wound healing. This work opens avenues for exploring the potential of cold plasma in surgical procedures [16].

Hyun-Jin Kim et al.’s “Analyses of the Chemical Composition of Plasma-Activated Water” shed light on the unique chemical compositions of plasma-activated water (PAW). The study suggests that PAW, with its antibacterial properties, could serve as a novel vaginal cleanser, offering protection against pathogens while preserving beneficial bacteria [17].

The “Synergistic antimicrobial effect of cold atmospheric plasma and redox-active nanoparticles” is explored by Artem M. Ermakov et al. In their study, the combination of cold argon plasma and metal oxide nanoparticles demonstrates a powerful inhibitory effect on bacterial growth, providing a potential avenue for antimicrobial treatments [18].

Dennis Feibel et al.’s research on the “Gas Flow-Dependent Modification of Plasma Chemistry in μAPP Jet-Generated Cold Atmospheric Plasma” highlights the importance of modulating gas flow for therapeutic use. Their findings suggest that adjusting the gas flow in micro-scaled atmospheric pressure plasma jets could influence plasma chemistry and optimize biological outcomes, paving the way for tailored clinical applications [19].

Mahsa Bagheri et al.’s preclinical evaluation, “Can Cold Atmospheric Plasma Be Used for Infection Control in Burns? A Preclinical Evaluation”, investigates the use of cold atmospheric plasma for infection control in burns. The study reveals that CAP is effective against Pseudomonas aeruginosa, a common infectious agent in burn wounds. While CAP’s efficacy is lower than that of some conventional treatments, its potential role in burn wound management is underscored [20].

Madline P. Gund et al.’s “Effects of Cold Atmospheric Plasma Pre-Treatment of Titanium on the Biological Activity of Primary Human Gingival Fibroblasts” explores the benefits of CAP treatment on titanium surfaces. The study suggests that CAP-treated titanium exhibits enhanced fibroblast coverage without altering biological behavior, offering insights into potential applications in implantology [21].

The second contribution by the research group, “Cold Atmospheric Plasma Improves the Colonization of Titanium with Primary Human Osteoblasts: An In Vitro Study”, by Madline P. Gund et al., investigates the effects of cold atmospheric plasma (CAP) treatment on titanium surfaces and its subsequent impact on primary human osteoblast colonization. The findings contribute to the growing body of evidence supporting the utility of cold atmospheric plasmas in biomedical applications, particularly in enhancing osteoblast colonization on implant surfaces [21].

Andreas Nitsch et al.’s investigation into the “Selective Effects of Cold Atmospheric Plasma on Bone Sarcoma Cells and Human Osteoblasts” provides crucial insights into the differential impact of CAP on malignant and non-malignant bone cells. The selective effect of CAP on sarcoma cells suggests its potential clinical application in oncology [22].

Finally, in their contribution, “Enrichment of Bone Tissue with Antibacterially Effective Amounts of Nitric Oxide Derivatives by Treatment with Dielectric Barrier Discharge Plasmas Optimized for Nitrogen Oxide Chemistry”, Dennis Feibel et al. enrich bone tissue with antibacterially effective amounts of nitric oxide derivatives using dielectric barrier discharge plasmas. This innovative approach presents a potential strategy for combating bacterial complications during bone healing [23].

The diverse studies presented in this Special Issue collectively underscore the transformative potential of plasma applications in biomedicine. While much progress has been made, these findings also highlight the need for continued research to fully unlock the therapeutic benefits and mechanisms of action underlying cold atmospheric plasmas and plasma-activated liquids.

We extend our gratitude to all the authors who have contributed to this Special Issue, providing valuable insights and advancing our understanding of the exciting and rapidly evolving field of plasma applications in biomedicine.
